# Innovative technologies and workplace collaborations in the energy sector based in the United Arab Emirates

**DOI:** 10.3389/frai.2026.1798647

**Published:** 2026-04-22

**Authors:** Jack Charles Boath, Sayed Abdul Majid Gilani, Tamaralaiyefa Harold Tiemo, Ansarullah Tantry

**Affiliations:** 1 Kings Business School UAE, Dubai, United Arab Emirates; 2 School of Management, Birmingham City University, Birmingham, United Kingdom; 3 Global Banking School, London, United Kingdom; 4 Fatima College of Health Sciences, Abu Dhabi, United Arab Emirates

**Keywords:** energy sector, innovative technologies, sustainability, UAE, workplace collaborations

## Abstract

**Introduction:**

The UAE energy sector is navigating digital transformation mandates such as the UAE AI Strategy 2031 and Net Zero commitments, with technologies like AI, IoT and cloud computing creating new avenues for real-time coordination, data-driven decision-making and cross-functional collaboration. These oppor tunities are tempered by challenges of organisational readiness, cultural iner tia and technological integration. Yet, research on innovative practices in the UAE energy context remains limited. Therefore, this study investigates the role of AI, IoT and cloud computing in shaping workplace collaboration in the UAE energy sector.

**Methods:**

An explanatory sequential mixed-methods design was adopted which involved Phase 1 (15 October, 2024–31 January, 2025) interviews with 15 professionals in operations, IT and leadership roles from major energy companies, analysed via thematic analysis. Phase 2 (15 February, 2025–15 May, 2025) distributed a survey to a broader sample, yielding 115 valid responses, which were analysed quan titatively. The study is primarily grounded in the Unified Theory of Acceptance and Use of Technology (UTAUT), with the Technology Acceptance Model (TAM), Resource-Based View (RBV) and Actor-Network Theory (ANT) serving as supporting interpretive lenses.

**Results:**

Findings show that AI, IoT, and cloud platforms enhance collaboration, especially in remote coordination and predictive decision sup port, but adoption is hindered by resistance to change, fragmented systems and uneven digital literacy.

**Discussion:**

Practical implications include modular rollouts, digital maturity audits and AI onboarding programs. Policy recommendations include national collaboration standards, KPI integration and incentives for joint innova tion projects.

## 1 Introduction

The UAE energy sector remains a central pillar of the United Arab Emirates national economy, contributing significantly to GDP and export revenues (UAE Ministry of Energy and Infrastructure, 2024) and continuing to shape the country’s economic development, industrial capacity and geopolitical influence. Hydrocarbon resources have historically driven national prosperity, forming the foundation of the UAE’s economic growth and global energy presence. Major national entities such as the Abu Dhabi National Oil Company (ADNOC) have undertaken significant investment programmes aimed at modernising operations and accelerating digital transformation across upstream, midstream and downstream activities. These initiatives reflect a broader strategic objective to maintain competitiveness in global energy markets while simultaneously advancing efficiency, operational resilience and sustainability objectives aligned with the UAE’s long term development strategies.

Central to this transformation is the integration of advanced digital technologies across energy operations. Artificial Intelligence (AI), the Internet of Things (IoT) and cloud computing have become key enabling tools supporting the digitalisation of industrial infrastructure. AI systems are increasingly applied to predictive maintenance, operational optimisation and large scale data analytics derived from production assets and energy networks (Aguiar-Hernandez and Breetz, 2024; Alami, 2023; Bojarajan et al., 2024). IoT technologies allow interconnected sensors and equipment to transmit real time operational data, improving situational awareness and decision making across geographically distributed facilities (Al-Hajri et al., 2024). Cloud computing further enables secure storage, large scale processing and collaborative access to operational information across organisations and locations, strengthening coordination between technical teams, regulators and management structures (Fasi, 2024; Currie et al., 2024).

These technologies extend beyond improvements in operational efficiency and increasingly influence organisational structures and communication processes within complex industrial environments. In the UAE energy sector, where projects often involve multidisciplinary teams spanning engineering, operations, information technology and regulatory oversight, digital platforms facilitate collaboration between actors operating across multiple locations and organisational boundaries. As the industry becomes increasingly data driven and interconnected, effective collaboration has become a critical capability for maintaining operational performance and organisational adaptability.

Despite the strategic importance of digital transformation in the UAE energy sector, several challenges continue to constrain the effective adoption and integration of advanced technologies. Cybersecurity risks remain a significant concern as increasing digital connectivity exposes critical infrastructure to potential cyber threats (Fasi, 2024; Mühlburger and Krumay, 2023). Workforce capability also represents a persistent barrier, as the operation and maintenance of advanced digital systems require specialised technical skills that are not yet widely available across the sector (Crupi and Schiliro, 2023; Al-Hajri et al., 2024).

Legacy infrastructure, organisational resistance to change and the complexity of integrating new technologies into existing operational systems further complicate the implementation of digital innovation initiatives (Bojarajan et al., 2024; Osmundsen and Bygstad, 2021).

While existing research has examined digital transformation within the global energy industry, comparatively limited attention has been given to the organisational implications of these technologies within the UAE context. In particular, empirical studies examining how AI, IoT and cloud computing influence collaborative practices among professionals working in UAE energy organisations remain scarce. Much of the existing literature focuses primarily on technical performance, operational efficiency or national energy policy, leaving the human, organisational and collaborative dynamics of technology adoption largely underexplored within the UAE energy sector.

This study addresses this gap by investigating how emerging digital technologies influence workplace collaboration within the UAE energy sector. Specifically, the research examines the role of AI, IoT and cloud computing in shaping collaborative practices among operational, technical and managerial personnel. By identifying both enabling factors and adoption barriers, the study aims to provide insights that support more effective integration of advanced technologies within complex industrial environments. The findings are expected to contribute to academic understanding of digital transformation in energy systems while offering practical implications for policymakers and industry leaders seeking to strengthen collaboration and innovation across the UAE energy landscape.

## 2 Literature review

### 2.1 Developments in the UAE energy sector

The UAE’s energy sector stands at the forefront of technological innovation in the Middle East, with AI, IoT and cloud computing transforming traditional operations into innovative, interconnected systems. Among the regional organisations, Dubai Electricity and Water Authority (DEWA) has realised the benefits of using AI and is applying machine learning algorithms to manage the electricity grid in the Emirate (Leal-Arcas, 2024). They use their AI systems to monitor consumption trends, weather and infrastructure to ascertain demand hitches, thus delivering service reliability.

Abu Dhabi National Oil Company (ADNOC) has revolutionised offshore operations through comprehensive IoT deployment in the petroleum sector. It has various sensors installed in the oil rigs in the Arabian Gulf that provide instantaneous details about equipment, the environment and production (Al-Hajri et al., 2024). This change from occasional physical inspections to constant digital supervision is radical in the UAE management of hydrocarbon resources. The IoT systems also play a role in sustainability issues, such as detecting methane leaks and, on the other hand, improving flaring activity efficiency.

Cloud computing has become the backbone of digital transformation for UAE utilities, with Etihad Water and Electricity’s (Etihad WE) migration to cloud-based analytics as a notable example (Mukhtar, 2024). The provider of the Northern Emirates has shifted from isolated, local systems to connected cloud systems to process customer consumption data, grid data and billing data.

Government policies have been instrumental in accelerating technological adoption across the UAE energy landscape. The UAE Energy Strategy 2050 envisions the country will have 50% of its total clean energy by mid-century (Tantry et al., 2025), which will encourage the investment of utilities and oil companies involved in managing their current and new assets through digitalisation.

Public-private partnerships have proven particularly effective in deploying advanced technologies at scale. The collaboration between Abu Dhabi National Energy Company (TAQA) and IBM illustrates this model, combining TAQA’s energy expertise with IBM’s AI capabilities to optimise renewable energy integration.

### 2.2 Review of global studies investigating innovation in the energy sector

Global research on innovation in the energy sector shows that digital technologies are increasingly reshaping operational efficiency, decision making and collaboration across both conventional and renewable energy systems. Studies from Europe, the Gulf region and other energy producing economies indicate that Artificial Intelligence (AI), the Internet of Things (IoT), cloud computing and related digital platforms are being deployed not only to improve technical performance but also to strengthen organisational responsiveness in complex operating environments.

Bojarajan et al. (2024) examined applications of AI in Scandinavian energy grids and reported substantial improvements in load forecasting, renewable integration and grid stability. Their study demonstrated how machine learning models supported the balancing of variable wind generation with hydropower resources, thereby improving operational coordination in highly digitised systems. In a similar vein, Aguiar-Hernandez and Breetz (2024) found that AI-enabled predictive analytics enhanced maintenance planning and reduced operational disruptions in energy infrastructure, particularly where large volumes of asset data were available for real time analysis. These findings suggest that AI contributes not only to technical optimisation but also to more coordinated decision making among engineering and operational teams.

Research on digital energy transformation in the Gulf region has produced comparable findings, although the emphasis often differs according to infrastructure maturity and policy priorities. In Saudi Arabia, studies on digitalisation within the energy and utilities sectors have highlighted the role of AI and IoT in supporting smart grid development, predictive maintenance and energy efficiency initiatives linked to Vision 2030. These studies suggest that digital technologies improve cross-functional coordination by enabling more integrated data flows between technical, operational and management units. In Qatar, research on smart energy systems and digital infrastructure has similarly emphasised the importance of cloud-enabled information sharing and connected monitoring systems in improving operational visibility and system responsiveness. Across these Gulf contexts, the common pattern is that digital innovation is increasingly associated with organisational integration as much as with technological capability.

Within the UAE, the literature shows growing interest in how digital tools are being deployed across energy operations, although much of the evidence remains focused on implementation outcomes rather than organisational effects. Al-Hajri et al. (2024) examined AI implementation within Dubai Electricity and Water Authority and reported that shared digital dashboards across generation, transmission and distribution functions enhanced coordination by providing a unified operational interface. This study is particularly relevant because it indicates that digital platforms can support collaboration across previously fragmented units. Fasi (2024), in contrast, examined blockchain applications in UAE energy trading and found that although the technology improved transparency in power purchase agreements and renewable energy certificate transactions, workforce readiness and digital capability limitations constrained its implementation. This suggests that technological potential alone is insufficient where organisational preparedness remains weak.

Additional international studies reinforce the importance of human and organisational factors in digital energy transitions. Currie et al. (2024) showed that cloud computing platforms support large scale data integration and shared access to operational information, enabling geographically dispersed teams to collaborate more effectively. Butler et al. (2023) argued that digital transformation in energy systems can contribute to resilience and sustainability, but only where organisational structures are capable of adapting to new data driven modes of working. Osmundsen and Bygstad (2021) similarly found that digital transformation is often hindered by resistance to change, fragmented legacy systems and weak alignment between technological implementation and organisational routines. Mühlburger and Krumay (2023) further identified cybersecurity concerns as a major barrier to digital adoption in critical infrastructure, with implications for trust, communication and system integration across energy organisations.

Taken together, these studies indicate that innovation in the energy sector is not solely a matter of introducing new technologies. Rather, the success of digital transformation depends on how technologies are embedded within organisational systems, how employees interact with them and how institutional structures support or constrain collaboration. Although global and regional research increasingly recognises the operational value of AI, IoT and cloud computing, empirical work examining their combined influence on workplace collaboration remains limited, particularly within UAE energy organisations. This gap justifies the present study’s focus on the human and organisational dimensions of digital innovation in the UAE energy sector.

#### 2.2.1 Theoretical integration and conceptual framing

This study is primarily anchored in the Unified Theory of Acceptance and Use of Technology (UTAUT), supported by the Technology Acceptance Model (TAM), the Resource-Based View (RBV) and Actor-Network Theory (ANT). These theoretical perspectives were selected because each explains a different but complementary dimension of technology adoption and collaboration in the energy sector.

UTAUT provides the principal explanatory framework for understanding behavioural intention and actual technology use. Its core constructs, namely performance expectancy, effort expectancy, social influence and facilitating conditions, are useful in examining why employees in the UAE energy sector may accept or resist AI, IoT and cloud-based systems. UTAUT is particularly relevant because it also incorporates moderators such as age, gender, experience and voluntariness of use, allowing the study to assess whether adoption patterns differ across employee groups.

TAM complements UTAUT by offering a more focused explanation of user perceptions through perceived usefulness and perceived ease of use. While UTAUT provides a broader model of acceptance, TAM helps to clarify how individuals evaluate the practical value and usability of specific technologies in everyday operational settings. In this study, TAM strengthens the interpretation of employee attitudes towards digital platforms used for communication, monitoring and data sharing.

RBV extends the analysis beyond individual perception by positioning digital technologies as strategic organisational resources. From this perspective, AI capability, IoT connectivity, cloud infrastructure and employee digital competence may be understood as valuable resources that can enhance organisational performance and collaborative capacity when effectively integrated. RBV therefore supports the argument that technology adoption is not only a behavioural issue but also a matter of institutional capability and competitive advantage.

ANT contributes a further layer by conceptualising collaboration as the outcome of interactions within a socio-technical network composed of human and non-human actors. In the context of this study, employees, managers, digital platforms, sensors, dashboards and organisational routines all function as actors within a network that shapes how collaboration occurs. ANT is particularly useful for interpreting how technologies do not merely support work but actively restructure communication patterns, authority relationships and operational practices.

Taken together, the four theories form an integrated explanatory structure. UTAUT and TAM explain why employees adopt and use technologies. RBV explains why organisations invest in and depend upon these technologies as strategic resources. ANT explains how the interaction between people, systems and processes reshapes collaboration in practice. Their integration allows the study to move beyond a narrow technology acceptance model and address the broader organisational and relational implications of digital transformation in the UAE energy sector.

**Table:** 

Theory	Primary focus	Contribution to this study
UTAUT	Behavioural intention and technology use	Explains employee acceptance of AI, IoT and cloud systems through performance expectancy, effort expectancy, social influence and facilitating conditions
TAM	Perceived usefulness and ease of use	Clarifies how employees evaluate the practical value and usability of digital technologies
RBV	Strategic organisational resources and capabilities	Interprets digital infrastructure and workforce capability as resources that support collaboration and performance
ANT	Socio-technical networks	Explains how human and non-human actors interact to shape communication, coordination and collaborative practices

#### 2.2.2 Operationalisation of the conceptual framework

**Figure 1:**
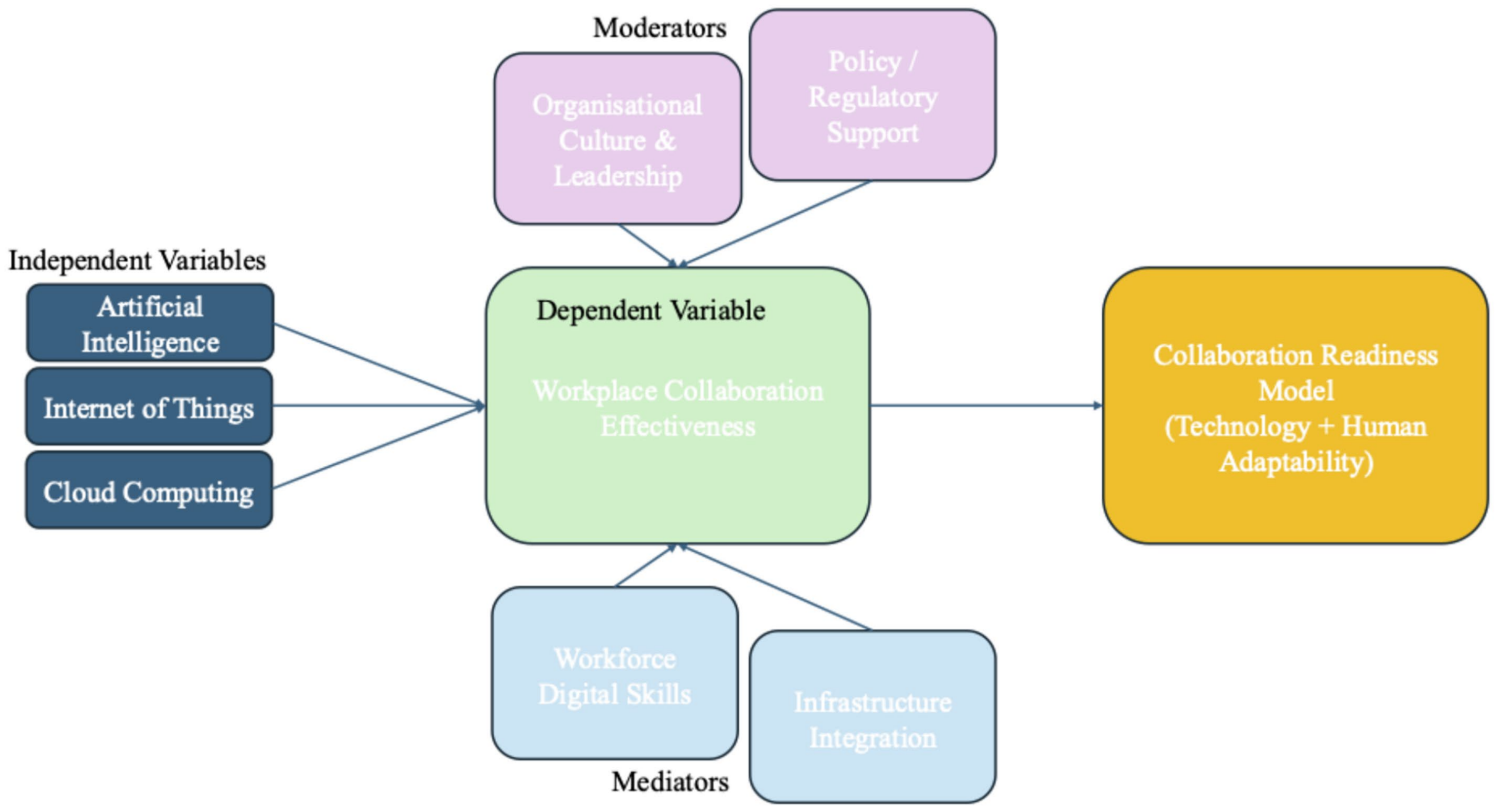
UTAUT-centred conceptual framework for innovation technologies and workplace collaboration in the UAE energy sector.

Figure 1 presents the conceptual framework guiding the study. The framework positions AI, IoT and cloud computing as the principal independent variables influencing workplace collaboration in the UAE energy sector. Workplace collaboration functions as the dependent variable and is understood in terms of communication quality, coordination efficiency, information sharing and cross-functional teamwork.

The framework also incorporates mediating and moderating factors. Perceived usefulness, perceived ease of use and behavioural intention were treated as mediating constructs derived primarily from TAM and UTAUT, reflecting the process through which technology characteristics influence collaborative outcomes. In practical terms, these were operationalised through survey items that measured the extent to which respondents believed digital technologies improved work effectiveness, were easy to use and encouraged continued use in professional tasks.

Moderating variables included age, professional experience, job role and organisational support conditions. These were incorporated to assess whether the relationship between technology adoption and collaboration differed across demographic and workplace contexts. Facilitating conditions, management support and digital skills were also examined as contextual enablers that could strengthen or weaken the effect of technology use on collaboration.

In the qualitative phase, these constructs were explored through interview questions focused on how employees experienced digital tools in practice, what barriers they encountered and how such technologies affected coordination across teams. In the quantitative phase, the same concepts were translated into measurable indicators within the survey instrument. This alignment between qualitative themes, theoretical constructs and survey variables ensured that the conceptual framework was not merely illustrative but directly operationalised within the study design.

### 2.3 Research gap

A review of existing studies reveals several key gaps in understanding the UAE’s energy sector transformation. Although cloud computing underpins AI and IoT systems, its specific role in enabling collaboration has received limited scholarly attention. Most research treats cloud infrastructure as a background enabler rather than analysing how different architectures, whether public, private or hybrid and vendor strategies influence collaboration outcomes in UAE energy organisations. This omission is significant given the nation’s reliance on cloud platforms and the unique data sovereignty and compliance requirements of the Gulf region. Further work must determine whether international cloud providers can satisfy local security expectations or if UAE-based solutions are more effective for mission-critical operations.

Another critical gap concerns organisational culture and workforce adaptation. While earlier studies acknowledge resistance to technological change, few examine its root causes or mitigation strategies within Emirati energy firms. The interplay between Emiratisation policies and digital transformation remains underexplored, particularly how workforce nationalisation goals may conflict with the immediate skill demands of advanced technologies. Broader research is needed on how traditional engineering mindsets interact with data-driven and AI-supported decision-making models.

Integration challenges also remain under-researched. Existing literature tends to focus on renewable pilot projects or isolated technology deployments rather than large-scale integration of AI, IoT and cloud systems across legacy hydrocarbon assets and new renewable infrastructures. Consequently, there is little understanding of how UAE energy companies can achieve unified digital ecosystems spanning diverse operational portfolios.

From a methodological standpoint, most UAE-based studies rely on qualitative case designs, limiting generalisability and external validity. Quantitative or longitudinal investigations that evaluate the sustained effects of technology adoption on collaboration and productivity are scarce. Moreover, current research often privileges managerial perspectives, neglecting frontline worker experiences that could offer practical insights into technology use.

Geographically, studies remain concentrated in Abu Dhabi and Dubai, leaving smaller Emirates underrepresented. Comparative analyses across Gulf states, particularly Oman, Qatar and Saudi Arabia, are also limited, restricting understanding of regional factors shaping adoption patterns.

Finally, research attention is uneven across technologies. AI studies emphasise generation and grid management, with little focus on AI’s contribution to collaborative work processes. IoT research remains hardware-centric, while blockchain analysis seldom extends beyond conceptual pilots. Emerging topics such as digital twins, quantum computing, generative AI, and cybersecurity within distributed energy networks are virtually absent from the UAE discourse despite their prominence in global energy innovation. Addressing these gaps is essential for developing a comprehensive understanding of how technological integration and collaboration co-evolve within the UAE’s rapidly modernising energy landscape.

### 2.4 Research objectives

The research objectives (ROs) for this study are outlined below.

*RO1:* To the role AI, IoT and cloud computing play in building better collaboration within the UAE energy sector. The evaluation targets how these technologies impact information exchange, communication and team cooperation in various departments and sections.

*RO2:* To evaluate organisational obstacles preventing their adoption within the energy industry. The energy sector faces barriers from staff reluctance to implement new approaches, security threats to data networks and problems with outdated system infrastructure. Recognising these challenges will be a foundation for creating practical solutions for their resolution.

*RO3:* To examine the impact of these technologies on operating efficiency, the development of better decisions and the evaluation of organisational performance. The research aims to verify if deploying technology-based collaboration generates increased performance while generating financial savings and achieving sustainability.

*RO4:* To investigate how innovative technology collaboration advances energy sustainability targets in the UAE. The study evaluates how better collaboration enables the adoption of renewable energy systems while lowering carbon pollution.

Collaboration effectiveness is conceptualised in this study as the extent to which digital technologies enable coordination, information sharing and joint decision-making across organisational units. Prior research has identified collaboration as a critical outcome of digital transformation, particularly in complex and data-intensive industries where interdependent teams must operate across functional and geographical boundaries (Nambisan et al., 2017). Digital platforms, cloud systems and IoT-enabled data integration have been shown to enhance communication efficiency, knowledge sharing and collective problem-solving, thereby improving collaborative performance within organisations (Porter and Heppelmann, 2014; Mubarak and Petraite, 2020). In this context, collaboration effectiveness is treated as a measurable organisational outcome influenced by the adoption of AI, IoT and cloud computing technologies.

### 2.5 Research hypotheses

The research hypotheses (H) are outlined below.

*H1:* The adoption of AI positively influences workplace collaboration by enhancing real-time decision-making and coordination.

*H2:* The implementation of IoT systems positively influences workplace collaboration by enabling data integration across departments.

H3: The use of cloud computing platforms positively influences workplace collaboration by supporting remote accessibility and cross-functional knowledge sharing.

*H4*: Organisational readiness and digital maturity moderate the relationship between innovation technologies (AI, IoT, Cloud) and collaboration effectiveness.

*H5:* Generational resistance and uneven digital literacy negatively moderate the relationship between innovation technologies and collaboration outcomes.

*H6:* The integration of AI, IoT and Cloud computing fosters the emergence of new forms of cross-organisational collaboration networks that extend beyond traditional hierarchical structures in the UAE energy Sector.

The operationalisation of these hypotheses through semi-structured interviews and survey instruments is detailed in Section 3.0 (Methodology), with full instruments and supporting materials provided in Appendices F–H in Supplementary material.

### 2.6 Conceptual framework

This study is primarily grounded in the UTAUT to explain the adoption and use of AI, IoT and cloud platforms in organisational settings. UTAUT’s core constructs: performance expectancy, effort expectancy, social influence and facilitating conditions, map directly onto the collaboration outcomes investigated here. TAM is retained as a conceptual precursor (via perceived usefulness/ease of use). RBV and ANT are used as supporting lenses: RBV to interpret collaboration as a strategic capability and ANT to illuminate socio-technical interactions among people, policies and technologies in the UAE energy context.

The Resource-Based View (RBV) offers a strategic perspective on how AI and IoT create sustainable competitive advantages for UAE energy companies through enhanced collaboration (Khalifa et al., 2025). The technologies are supported by a cloud computing infrastructure that affords convenient and powerful shared access to data for geographically distributed collaborating teams, which gives an organisation beyond individual technologies. RBV can explain why implementing the identical AI software system to competitors does not mean similar performance – the key competitive advantage lies in how such technologies are applied, deployed and applied jointly within the context of each firm’s operations and strategic goals in the energy sector.

Actor-network theory (ANT) provides a radically different lens for examining human-technology interaction in UAE energy projects, rejecting the traditional separation between technical and social elements. Due to ANT, following these connections and determining whether the interplay is conflicting or complementary when applying technology is easier. In the UAE context, it explains how such a policy as the Energy Strategy 2050 is a non-human agent that reconfigures the role of energy providers, technology suppliers, regulators and consumers (Tantry et al., 2025). In discussing IoT ecosystems, the theory is most helpful due to its focus on horizontal relationships, which allows the identification of how the effectiveness of a technological solution depends on sensors, communication protocols, data standards and maintenance procedures. When these networks are mapped, energy companies can also prepare for potential problems within implementation and create better socio-technical systems.

UTAUT extends TAM by incorporating social and organisational determinants of use in the UAE’s distinctive cultural context (Bouteraa et al., 2023). Accordingly, UTAUT is adopted as the principal framework, while TAM is treated as its conceptual precursor. RBV and ANT serve as supporting interpretive perspectives — RBV offering a strategic lens on capability development, and ANT providing insight into the socio-technical networks shaping technology integration in the UAE energy sector. The review of innovation adoption theories has informed the development of a UTAUT-centred conceptual framework for this study, which is illustrated in Figure 1.

In Figure 1, the independent variables comprise three innovation technologies, which are Artificial Intelligence, the Internet of Things and cloud computing. Each of these is hypothesised to influence the dependent variable, workplace collaboration effectiveness, positively. Two sets of contextual factors shape the relationship. Firstly, moderators such as organisational culture and leadership, along with policy and regulatory support, determine the extent to which technology adoptions translate into effective collaboration.

Second, mediators, including workforce digital skills and infrastructure integrations, provide the operational capacities for collaboration outcomes to be able to materialise. Finally, the framework converges on a proposed Collaboration Readiness Model, which integrates both technological and human adaptability dimensions, reflecting the combined impact of these factors on sustainable collaboration in the UAE energy sector.

### 2.7 Research questions

The research questions (RQs) for the study are outlined below.

*RQ1:* How do AI, IoT and cloud computing support collaboration within the UAE’s energy sector?

*RQ2:* What are the barriers to the adoption of AI, IoT and cloud computing in the UAE's energy sector?

*RQ3:* In what ways do innovative technologies contribute to operational efficiency and performance in the UAE energy sector?

*RQ4:* How does technology-driven collaboration support the UAE’s sustainability and energy goals?

## 3 Methodology

This study adopted an explanatory sequential mixed methods design to investigate how innovative technologies influence collaboration in the UAE energy sector. The approach combined qualitative and quantitative methods to provide both depth of understanding and broader generalisability of findings. In this design, qualitative exploration preceded quantitative testing, allowing insights derived from expert interviews to inform the development of the survey instrument used in the second phase.

Participants were identified through professional networks, industry contacts and publicly available organisational directories within major UAE energy institutions. Potential participants were contacted via professional email and LinkedIn invitations explaining the purpose of the research and requesting voluntary participation. Interviews were conducted primarily through secure virtual meeting platforms due to scheduling and geographical considerations, although participants were offered the option of in-person meetings where feasible. Each interview lasted approximately 40–60 min and followed the structured interview guide presented in Appendix G in Supplementary material.

### 3.1 Phase 1 (Qualitative study)

Semi-structured interviews were conducted with key stakeholders from major UAE energy organisations, including ADNOC, DEWA and TAQA. Primary data collection took place between 30 October 2024 and 31 January 2025. Prior to the main interviews, the interview protocol was pilot tested with five participants between 10 September and 20 September 2024. The pilot study assessed the clarity, relevance and sequencing of the interview questions. Feedback from the pilot participants indicated that some questions required simplified wording and clearer phrasing, resulting in minor revisions to improve participant understanding.

Participants were selected using a criterion-based purposive sampling strategy, targeting individuals with direct operational or managerial involvement in Artificial Intelligence, Internet of Things and cloud computing initiatives. Purposive sampling was considered appropriate because the study required participants with specialised experience of digital transformation within energy organisations. This deliberate sampling ensured that all participants possessed relevant expertise and were actively involved in technology implementation or management within their organisations.

Recruitment continued until thematic saturation was achieved. Fifteen usable interviews were obtained from an initial pool of 24 invited participants. The participants represented a range of operational, technical and leadership roles across the UAE energy sector, ensuring that multiple organisational perspectives on digital technology adoption and collaboration were captured.

### 3.2 Phase 2 (Quantitative study)

Survey participants were recruited using professional networks, industry mailing lists and referrals from Phase 1 participants. Invitations were distributed electronically via email and professional platforms, with a secure survey link provided to ensure confidentiality and voluntary participation. Participation was restricted to professionals currently working within the UAE energy sector to ensure relevance to the research objectives.

Insights generated from the qualitative phase informed the development of a structured survey questionnaire designed to examine the prevalence of the identified themes across a broader population. The survey instrument was pilot tested with 10 participants between 4 February and 14 February 2025 to evaluate question clarity, structure and completion time. Feedback from the pilot participants resulted in minor adjustments to wording and question sequencing to ensure that the survey items accurately reflected the constructs derived from the qualitative findings and the theoretical framework.

The final survey was administered between 15 February 2025 and 15 May 2025. A stratified purposive sampling approach, complemented by voluntary response participation, was used to obtain representation across different organisation types within the UAE energy sector, including utilities, oil and gas and renewable energy organisations. The sampling strategy also aimed to capture diversity across professional hierarchies, including executive, managerial and technical roles.

A total of 212 surveys were distributed, from which 115 usable responses were obtained, representing a response rate of approximately 54 percent. This sample size is considered adequate for exploratory statistical analysis in organisational and technology adoption research, particularly within specialised professional populations where access to respondents is limited. The sample size also allowed for meaningful examination of relationships between technology adoption constructs and collaboration outcomes.

### 3.3 Data analysis

Qualitative data were analysed using thematic analysis. Coding combined deductive and inductive approaches. *A priori* codes were derived from constructs associated with the Unified Theory of Acceptance and Use of Technology, including performance expectancy, effort expectancy, social influence and facilitating conditions, supported by constructs from the Technology Acceptance Model such as perceived usefulness and perceived ease of use. Additional interpretive insights were considered through the Resource-Based View, particularly where technology capabilities were discussed as organisational resources. Emergent coding was also applied to identify new themes that arose from the data, including generational differences, cultural influences and concerns relating to cloud security.

The statistical analysis was conducted using standard regression and correlation procedures commonly applied in technology adoption research. Model assumptions and statistical outputs were reviewed to ensure internal consistency between correlation, regression coefficients and model significance values. The analysis followed established quantitative research practices used in UTAUT-based studies, allowing reliable interpretation of the relationships between technology adoption variables and collaboration outcomes.

A manual analysis process was conducted in Microsoft Excel in order to maintain close engagement with the qualitative data and ensure transparency in the coding process. Excel was used to organise transcript excerpts, apply and refine codes and conduct pattern and frequency analysis. Comparative analysis across ADNOC, DEWA and TAQA enabled the identification of both shared trends and organisation-specific differences, including variations in digital maturity, collaboration practices and resistance to technological change.

Quantitative data were analysed using statistical techniques designed to examine relationships between technology adoption constructs and workplace collaboration outcomes. The survey instrument included items derived from UTAUT and TAM constructs, allowing measurement of perceived usefulness, ease of use, facilitating conditions and behavioural intention in relation to collaborative practices.

To assess reliability, internal consistency of the survey constructs was evaluated using Cronbach’s alpha. The resulting reliability coefficients exceeded the commonly accepted threshold of 0.70, indicating satisfactory internal consistency among the measurement items. Construct validity was supported through alignment between the survey items, theoretical constructs and themes identified during the qualitative phase.

Integration of qualitative and quantitative findings occurred during the interpretation stage of the research. This integration allowed triangulation of results and strengthened the validity of the study by comparing patterns observed in the qualitative interviews with those identified in the survey data.

Manual coding was performed on anonymised transcript excerpts (Appendix A in Supplementary material), and excerpts from the coding framework and coding sheets are presented in Appendices D,E in Supplementary material, respectively. The thematic development process is illustrated in the thematic map (Appendix C in Supplementary material), while a summary of interview responses is provided in Appendix B in Supplementary material.

### 3.4 Ethical considerations

The study adhered to the ethical policy of King’s Business School. Full ethical clearance was obtained prior to data collection on 16 October 2024 under ethics approval reference KBS_16/10/2024-Boath_J. All participants provided informed consent before participation, and all data were stored and managed securely in accordance with institutional requirements for confidentiality and research integrity.

## 4 Findings

This section integrates the qualitative and quantitative results obtained through the sequential mixed-methods design. The findings are structured to first illustrate the qualitative themes (Phase 1) and then validate them through quantitative evidence (Phase 2).

### 4.1 Phase 1

The initial phase of analysis identified recurring themes from the semi-structured interviews, which were refined through iterative coding and constant comparison. These thematic outcomes represent the most prominent dimensions of how innovative technologies influence collaboration within UAE energy organisations.

**Figure 2:**
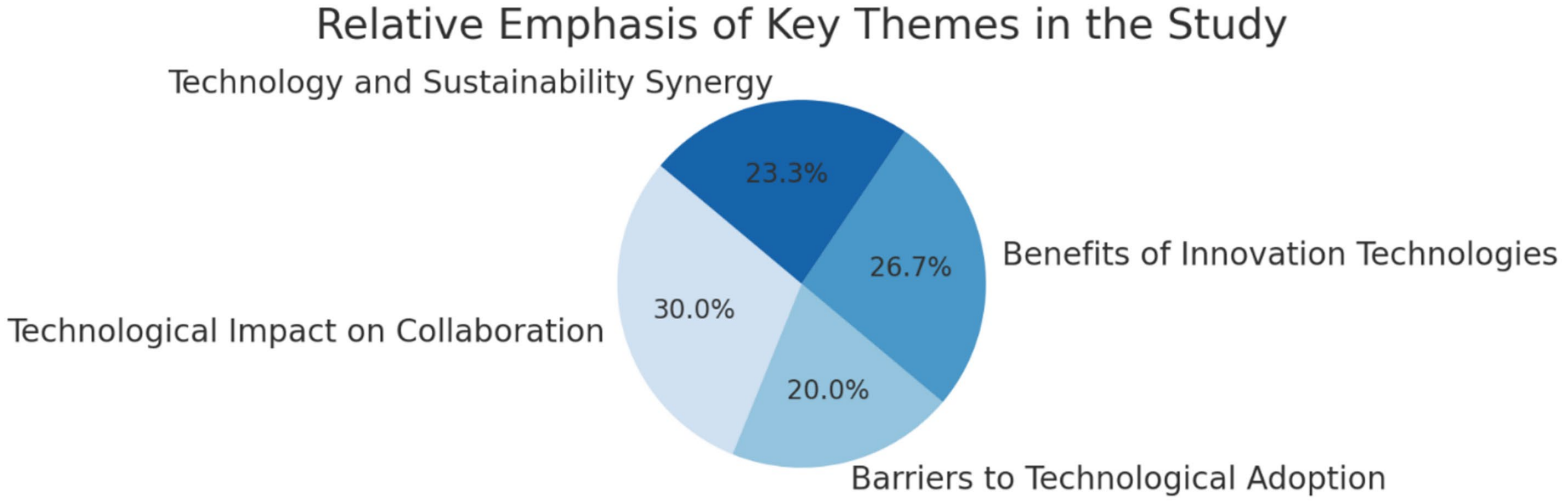
Perceived impact of themes (ranked by percentage response).

Table 1 summarises the four dominant themes derived from the qualitative dataset, while Figure 2 illustrates the perceived relative impact of these themes as expressed by both interviewees and survey respondents. Together, they provide a structured overview of the qualitative foundation that informed the subsequent quantitative phase.

**Table 1:** Key themes summary.

#	Theme	Key insights
1	Technological impact on collaboration	AI, IoT and cloud computing improve cross-functional team coordination, decision-making and digital workflows.
2	Barriers to technological adoption	Cybersecurity risks, legacy systems and resistance to change hinder the adoption and integration of technologies.
3	Benefits of innovation technologies	Enhanced operational efficiency, faster decision-making and improved organisational agility.
4	Technology and sustainability synergy	Technologies support sustainability monitoring, renewable energy integration and alignment with national strategies.

The four themes collectively demonstrate that innovative technologies are not merely operational tools but catalysts for inter-departmental collaboration and strategic alignment.

Theme 1 highlights how digital platforms enable real-time communication and coordination across geographically dispersed teams.

“Cloud-based dashboards have changed how we coordinate offshore maintenance; we can see updates instantly rather than waiting for reports.” (Operations Engineer, ADNOC).

“AI-driven predictive tools help our teams prioritise safety-critical tasks faster and with fewer communication delays.” (IT Manager, TAQA).

Theme 2 reflects organisational and behavioural resistance as major obstacles to technological uptake, often linked to cybersecurity concerns and outdated infrastructure.

“We still have teams that don’t trust data stored on the cloud; they print everything.” (Senior Supervisor, DEWA).

“The biggest issue isn’t the technology; it’s changing the mindset of senior management used to legacy systems.” (Digital Transformation Lead, ADNOC).

Theme 3 captures the tangible benefits observed through increased efficiency and agility.

“IoT monitoring has reduced unplanned downtime by giving us early warnings before faults occur.” (Maintenance Engineer, TAQA).

“AI has allowed us to plan resources dynamically; we save time and improve coordination between departments.” (Project Manager, DEWA).

Finally, Theme 4 emphasises the growing alignment between technology adoption and national sustainability objectives, confirming a broader socio-economic dimension.

“We now track carbon intensity in real time through cloud systems integrated with IoT sensors.” (Sustainability Officer, ADNOC).

“AI and automation are not just about efficiency anymore — they directly support our Net Zero 2050 reporting targets.” (Energy Strategy Analyst, TAQA).

“The sustainability dashboards allow our engineering and environmental teams to work from the same real-time data, which makes reporting and compliance much easier.” (Environmental Engineer, DEWA).

Figure 2 visually represents how participants ranked these themes in terms of their perceived impact on collaboration, showing that technological impact was rated highest, followed by benefits of innovation technologies, then sustainability synergy, with barriers to technological adoption ranked lowest.

#### 4.1.1 Theme 1: Technological impact on workplace collaboration

This theme analysed the transformative impact of innovation technologies, AI, IoT and cloud computing, on workplace collaboration within the UAE’s energy sector. These technologies signify a significant shift to a more synchronised approach with the goals of data-centric cooperation and the nation’s vision for digital transformation and sustainable development.

The implementation of AI technologies promotes proactive collaboration. AI’s foresight can identify anomalies, plan and adapt well in advance, which entails collaboration across multiple departments. Within UAE-based energy corporations, AI technologies are used for analytics, predictive maintenance and demand forecasting, monitoring electricity supply on the grid and predicting system failures (Al-Hajri et al., 2024; Bojarajan et al., 2024). Such forecasts are not role-specific but trigger maintenance teams, operations, data analysts and change managers.

This common reliance on AI-derived insights has led to greater interconnected processes in a workflow. The predictive algorithms involved in grid optimisation offer actionable intelligence and necessitate team synchronisation around real-time data outputs for collective decision-making. Thus, AI’s predictive capabilities foster collaboration. The technology fundamentally alters decision-making from a hierarchical structure to a shared structure (Pirozzi, 2024). Moreover, AI enhances the precision and promptness of decisions by analysing real-time data (Erhueh et al., 2024).

Although AI assists in foresight and strategic cooperation, the IoT has changed coordination at the operational level through real-time data collection and visibility of systems. Al-Hajri et al. (2024) identified smart meters, remote sensors and monitoring drones as IoT technologies that ensure data continuity across the energy value chain from production facilities to distribution grids. These data streams are available to multiple users in different sections, thus creating shared operational awareness that facilitates collaboration. For instance, maintenance teams use IoT data to determine equipment issues, and the engineering department leverages the same data for performance analytics (Aguiar-Hernandez and Breetz, 2024).

IoT technologies resolve these issues via real-time communication with control centres from the field. According to Bahyan et al. (2024), IoT enhances system monitoring automation. These improvements improve the resiliency and responsiveness of an energy system. Decentralisation empowers responsive cross-functional team action and fosters swift decentralised responsibility.

The integration of AI and IoT is allowing data democratisation – stakeholders at different echelons of the organisation can access data, shared assets, dashboards and interactive decision-making tools in real time.

The UAE’s leading energy companies, such as ADNOC, DEWA and Etihad Water and Electricity (Etihad WE), implemented cloud-based systems to streamline collaboration between remote teams. Cloud-based dashboards that consolidate AI forecasts, IoT information and project timelines enable interdisciplinary pods to interact with the system concurrently. This digital collaboration environment increases alignment and enhances purposeful collaboration (Erhueh et al., 2024).

Cloud computing facilitates real-time collaborative decision-making and project execution. Team members from various departments can together modify schedules, upload reports or change project parameters. These functions are critical for projects with public-private participant collaborations, such as solar grid expansions or upgrades to offshore drilling. Thus, cloud-based systems enable energy organisations to cooperate vertically and laterally. Besides supporting internal collaboration, cloud systems also allow collaboration with external vendors, contractors and government regulators.

This functionality is crucial for national collaborative inter-institutional projects like the UAE Net Zero by 2050 Strategy and the UAE Energy Strategy 2050. Furthermore, cloud solutions offer scalability, which allows energy companies to meet increased demand and expand collaborative frameworks without system-overhaul restructuring.

Each technology, AI, IoT and cloud computing, offers specific advantages to collaboration in the workplace, but their integration provides even greater benefits. The combination of these technologies creates intelligent ecosystems that can interactively gather, process information, analyse (AI) and distribute (cloud computing) relevant data as well as perform the necessary collaborative functions. As an illustration, predictive maintenance, a frequent use case in the UAE’s energy sector, depends on IoT sensors to identify issues, uses AI models for urgency evaluation and employs cloud dashboards for notification dissemination (Poyyamozhi et al., 2024).

#### 4.1.2 Theme 2: Barriers to technological adoption

Although the energy sector of the UAE has made strides in adopting emerging technologies such as AI, the IoT and cloud computing, the investigation highlighted several remaining barriers that constrain the full potential of these technologies. The results from the interviews, combined with thematic analysis, underscored three prominent subthemes: cybersecurity risks, legacy infrastructure and organisational resistance to change.

Participants were concerned about cybersecurity risk due to higher levels of digitalisation. Implementing AI, IoT and cloud systems streamlines processes and amplifies the numerous touch points for data collection and access. However, such innovations raise the susceptibility of infrastructure systems to cyber threats. This concern is magnified in the energy sector, which is considered critical infrastructure in the context of UAE national security.

Dependence on modern infrastructures like smart grids, remote sensors, and cloud-based analytics indicates that disruption of a single element can compromise the entire network’s security (Mazhar et al., 2023). Interviewees indicated apprehensions that complete fusion of IoT and cloud systems, particularly those with real-time operational data streams, may risk exposing their enterprises to data leaks, service interruptions or even focused cyberattacks. Such fears are consistent with the results reported by Fasi (2024). The increased complexity of digital ecosystems involving AI-enhanced decision engines, IoT-enabled surveillance and cloud-hosted databases presents challenges that current cybersecurity protocols struggle to address.

Even though the UAE has invested heavily in regulatory safeguards and national cybersecurity infrastructure, including data localisation mandates and zero-trust architecture, many companies feel the pace of innovation outstrips the maturity of existing safeguards. For example, some organisations restrict access to predictive maintenance dashboards to specific roles due to cybersecurity policies, even though broader access would support faster and more coordinated decision-making.

Another significant barrier revealed through the analysis is the technological incompatibility between legacy systems and modern digital solutions. Numerous organisations still utilise hardware and software systems that do not interface with AI, IoT or cloud systems. Multiple participants emphasised that integrating IoT sensors with pre-existing SCADA systems results in fragmented systems rather than fully integrated ones. These challenges go beyond technical concerns and have organisational and financial impacts. Companies often require expensive middleware, bespoke APIs, or entire system overhauls to bridge the gap (Al-Hajri et al., 2024).

As highlighted by Bojarajan et al. (2024), companies are likely to limit their technological implementation to pilot projects because of the acute technical challenges. For example, specific units may have access to AI-based alerts for performance notifications on equipment. In addition, legacy systems contribute to poor real-time analytics and unnecessary duplication in data governance processes. This overload increases the burden on IT departments managing redundant systems, straining extra workloads and fiscally reallocating resources away from progressive initiatives. These primary IT systems warrant such fears because of the local financial expenditure, the accompanying business processes risks and the potential for service interruptions during steps.

Socio-cultural perspectives also hinder the adoption of technology. Organisational cultures primarily shape attitudes regarding the advancement and adoption of technologies. Respondents noted that staff, particularly those in senior or managerial roles, tend to shy away from utilising AI information and transitioning from paper-based systems to cloud-based systems. This mindset is the result of an intense fear of being technologically made redundant, a fierce resistance to new and oftentimes dull automated innovations thought to be devoid of human intelligence and a conviction that algorithms devoid of human reasoning cannot guide logical thought processes which aligns with Fasi (2024) and Crupi and Schiliro (2023) who observed that cultural resistance was one of the most prominent barriers to digital transformation in the energy sector.

This form of behavioural resistance is worsened by the varying levels of technological skills across age groups. Employees from younger generations tend to forecast new technologies as part of their functions because they are digital natives. Conversely, older employees often require intensive training and, in some cases, ongoing support to feel comfortable with new systems (Francis-Pettway, 2019).

A lack of communication often compounds resistance to change. When frontline employees are not consulted regarding the purpose and benefits of new technologies, they tend to view such initiatives as hostile, which illustrates the need for comprehensive change management that balances technological and social aspects of adopting new systems. Moreover, organisational culture shapes other areas, such as leaders and the governance structure. In many Emirati energy companies, decision-making remains centralised, with senior leadership dictating the pace and scope of digital initiatives. While this aligns strategic priorities with national agendas, it may inhibit bottom-up innovation and prolong the feedback cycles critical for effective technology implementation (Al-Hajri et al., 2024).

#### 4.1.3 Theme 3: Benefits of innovation technologies

Notwithstanding the difficulties of innovative technologies, the information gathered from this study indicates that AI, the IoT and cloud computing technologies have been beneficial at operational, managerial and strategic levels. These technologies facilitate collaboration at the workplace and are instrumental in improving organisational effectiveness, driving down costs, boosting agility and enhancing responsiveness. Three primary benefit categories emerged from the interview analysis and thematic coding: enhanced operational efficiency, more rapid decision-making and heightened organisational agility.

Participants repeatedly emphasised the considerable gains in operational efficiency after implementing AI, IoT and cloud technologies. The integration of these technologies facilitated automation of monotonous tasks, proactive maintenance and optimal resource utilisation, all of which increased productivity and efficiency in workflows. AI has streamlined energy operations, and its predictive capabilities have sharpened proactively (Bello et al., 2024). ADNOC and DEWA participants showcased how machine learning algorithms monitor equipment and energy grids, providing early warnings of potential failures. This predictive capability minimises downtime and emergency repair work, enabling more efficient scheduling and utilisation of technical personnel (Bojarajan et al., 2024; Al-Hajri et al., 2024).

Likewise, IoT devices such as smart meters and sensors have eliminated the guesswork in tracking performance metrics. These devices stream real-time operational data to central repositories, revealing valuable information regarding energy utilisation, equipment functionality and system irregularities, which helps departments pinpoint inefficiencies and remediate them promptly. As Al-Hajri et al. (2024) noted, the IoT has changed routine inspections to precision interventions, drastically improving the effectiveness and safety.

In addition, cloud computing enhances efficiency by providing storage and access to data in real time. With cloud platforms, all project participants can access shared data, dashboards and workflow tools that minimise duplication and improve accuracy (Egbuhuzor et al., 2024).

A further improvement noted is the speeding up of decision-making across various company levels. With AI analytics, IoT monitoring and cloud-based dashboards, organisations can now access a rich informational terrain, allowing for more confident and swift action relative to agile operational conditions. AI systems facilitate and enhance decision-making capabilities through predictive modelling and intelligent recommendations. AI systems provide real-time forecasts, performance simulations and risk assessments (Erhueh et al., 2024).

IoT technologies provide AI systems with the requisite constant data streams, thus bolstering such capabilities. Sensors within turbines, transformers and solar panels monitor minute shifts in their operational environment. This continuous feedback transforms decision-making into dynamic ones based on evidence. In addition, using cloud platforms allows different departments, such as operations, IT, safety and compliance, to access, share and work on the same dataset simultaneously (George, 2022). Cloud dashboards’ ability to draw information from disparate systems updates all users simultaneously, acting as a single source of truth, improving information visibility and ensuring swift collective response.

The most significant additional advantage noted pertains to the improvement in organisational agility, which is how energy firms can scale and transform operations with changing conditions. The enhanced responsiveness with which companies can manage unanticipated shifts in power generation, consumption and equipment failures is possible due to the dynamic evaluation of power, equipment and environmental parameters. Resilience can be enhanced through AI-enabled simulations that test multiple operational models and devise counter-response strategies for disruptive scenarios, servicing weather-enabled demand spikes and planned or unplanned outages (Al-Hajri et al., 2024).

Decentralised supervision and control are enabled through IoT devices, which enhance organisational agility (Bojarajan et al., 2024). The shift in decentralisation also alters the balance of power within teams.

Organisational agility is enhanced with cloud computing due to the cross-collaboration of different business units, thus providing elastic computing resources. For large-scale energy projects involving multiple vendors, government agencies and investors, cloud platforms serve as shared spaces for data exchange, progress tracking and real-time coordination. Additionally, cloud systems facilitate the scaling of operations. As energy projects grow, expanding solar operating capacity or deploying IoT sensors over new infrastructure, cloud services easily accommodate these changes without requiring major hardware or IT personnel reinvestments.

#### 4.1.4 Theme 4: Technology and sustainability synergy

AI and IoT technologies offer real-time monitoring and accurate tracking of key sustainability metrics (Villegas-Ch et al., 2024). Emissions, water consumption, energy usage and energy generation are some indicators being monitored. Modern power utilities have adopted proactive measures as a result of the implementation of AI and IoT systems. These systems provide real-time feedback, which enables companies to assess their operations and make the necessary adjustments for ongoing compliance with sustainability limits.

The IoT sensors facilitate the real-time monitoring of solar panels, power grids and cooling systems. This feature has proved crucial for companies trying to meet internal sustainability mandates and external frameworks like the UAE’s Green Agenda and National Climate Change Plan. Al-Hajri et al. (2024) demonstrated the importance of monitoring sensors for assessing carbon intensity in industrial processes. Moreover, Bojarajan et al. (2024) indicated that diagnostics powered through AI reduce energy waste by system performance optimisation. The findings of this study confirm these assertions since participants reported that the technologies assist in minimising non-essential energy expenditures, identifying wasteful devices and conducting environmental compliance audits more efficiently.

The integration of new age technologies facilitates the incorporation of renewable energy sources into the national energy grid. In the United Arab Emirates, integrating and managing solar, wind and hydrogen energy, AI, IoT and cloud computing are essential. Participants pointed out the impact of these novel technologies in managing the control of changing energy inputs, streamlining the utilisation of renewables, collaborating among multiple divisions and stakeholders in the coordinated energy transition and performing optimisation tasks. Systems based on IoT assist in monitoring the functioning of solar panels and wind turbines and give real-time information about outputs, efficiencies and environmental parameters (Karad and Thakur, 2021). These parameters reinforce dependability while addressing grid integration challenges of renewables, further improving the contribution of renewables to the grid reliability and stability. AI assists in forecasting demand and supply shifts, enabling grid managers to integrate renewable and conventional sources of electricity into the grid. Maintaining this equilibrium is vital to avert overproduction, underutilisation and grid overload; any of these scenarios would undermine the sustainability targets of the UAE Energy Strategy 2050.

The study found that collaboration via cloud platforms is critical for scaling renewable energy projects. The disciplines involved in solar farm construction, such as operations, finance, policy, grid integration and regulatory compliance, must interact.

A further critical takeaway from the research is national policy frameworks that support sustainability, such as the UAE Vision 2031, the National Energy Strategy 2050 and the strategic initiative of Net Zero by 2050. As highlighted by the study participants, the so-called innovation technologies fulfil operational and environmental functions and serve strategic compliance and reporting functions that help organisations monitor their performance vis-à-vis government benchmarks. Alami (2023) and Bojarajan et al. (2024) highlighted that digital innovation serves as a primary pillar of strategy implementation in the energy sector of the United Arab Emirates (UAE), thus confirming the earlier conclusions.

As the central repositories of all relevant data and documents on sustainability, cloud platforms are pivotal in enabling policy alignment. These systems help with internal reporting and provide external clarity, assisting organisations in informing regulators, investors and other global stakeholders. Furthermore, the capacity to provide information in real-time and at scale improves policy engagement. With IoT cloud frameworks, for example, AI enables energy firms to instantly evaluate their standing and modify operations in real-time upon establishing carbon targets and new green construction policies (Rane, 2023).

Participants had consistently highlighted that achieving large-scale sustainability outcomes, such as carbon neutrality, hydrogen integration and green infrastructure expansion, cannot be realised by individual organisations alone. Respondents pointed to a growing reliance on external collaboration with government bodies, technology providers and academic institutions to meet national energy and environmental targets. These collaborations were often described as technologically enabled, particularly through the use of shared cloud-based platforms. Examples illustrate how the cloud has become a neutral coordination layer, allowing data transparency and real-time task execution across organisational boundaries.

Many participants referenced specific cases, including solar farm development, hydrogen pilot programs and emissions reporting, where stakeholders from multiple sectors worked within a unified digital environment. These platforms provided synchronised dashboards for performance tracking, regulatory compliance updates and shared documentation, facilitating ongoing cooperation among otherwise separate entities.

This convergence reflects an evolving model of digitally enabled inter-organisational collaboration in the UAE energy sector. Rather than acting in isolation, companies are forming technology-driven alliances that rely on real-time data integration, cloud coordination and multi-party accessibility.

The qualitative findings reveal that innovative technologies, particularly AI, IoT and cloud computing, are redefining how collaboration occurs across the UAE energy sector. Participants consistently described a shift from traditional hierarchical coordination to agile, data-driven and digitally connected teams. AI was perceived as a key enabler of faster evidence-based decision-making, while IoT and cloud systems enhanced transparency, accessibility and cross-departmental alignment.

However, organisational resistance, legacy systems and uneven digital literacy continue to hinder full integration. These barriers were viewed less as technological constraints and more as cultural and structural challenges requiring leadership commitment and digital change management. Despite these limitations, respondents highlighted that technology-driven collaboration is accelerating alignment with national sustainability goals and operational resilience objectives.

Overall, the qualitative phase confirms that digital technologies act as both technical enablers and social catalysts, reshaping not only workflows but also communication norms, decision hierarchies and performance expectations. These insights informed the design of the quantitative survey, particularly in operationalising variables such as performance expectancy, effort expectancy, social influence and facilitating conditions in accordance with the UTAUT.

### 4.2 Phase 2

Following the qualitative phase, the quantitative analysis was explicitly designed to validate and statistically generalise the themes identified in Phase 1. The key qualitative themes, including technological impact, benefits of innovation technologies, adoption barriers and sustainability alignment, informed the development of the survey instrument and the selection of measurable variables. In particular, qualitative insights relating to performance improvements, usability, organisational support and behavioural responses were operationalised into constructs aligned with UTAUT and TAM.

This sequential approach ensured that the quantitative phase did not operate independently, but rather tested the prevalence and strength of relationships identified during the qualitative analysis. As such, the integration of both phases reflects an explanatory sequential mixed-methods design, enhancing the coherence, validity and theoretical consistency of the study.

The descriptive statistics in Table 2 outline the overall distribution of responses for the main study variables, collaboration effectiveness, technology integration, leadership support and digital literacy. Table 3 compares these variables to estimate their relative contribution to collaboration outcomes, based on manual tabulation and frequency analysis.

**Table 2:** Descriptive statistics (survey responses, *n* = 115).

Variable	Mean	SD	Min	Max
Collaboration effectiveness	3.9	0.8	2	5
Technology integration	4.1	0.7	2	5
Leadership support	3.8	0.9	1	5
Digital literacy	3.7	0.8	2	5

**Table 3:** Comparative patterns from manual tabulation.

Factor	% reporting positive effect	Approx. contribution to collaboration (manual estimate)
Technology integration	78%	15%
Leadership support	72%	12%
Digital literacy	69%	13%
Combined contribution	—	~40%

Table 4 (A) provides a demographic overview of the survey respondents, illustrating representation across organisation types, professional roles and levels of experience within the UAE energy sector.

**Table 4:** (A): Respondent demographic profile (*n* = 115).

Category	Distribution
Organisation type	Oil and gas (46%), utilities (32%), renewable energy (22%)
Role level	Executive (18%), managerial (39%), technical/operational (43%)
Experience	<5 years (21%), 5–10 years (34%), >10 years (45%)
Technology exposure	Direct involvement in AI/IoT/Cloud initiatives (68%), Indirect exposure (32%)

The descriptive statistics in Table 3 indicate that respondents generally expressed agreement regarding the positive role of digital technologies in workplace collaboration. On a five-point Likert scale, mean scores ranged from 3.7 to 4.1. A mean of 3.9 for collaboration effectiveness indicates moderate to strong agreement that digital technologies enhance team coordination and information sharing. Technology integration recorded the highest mean (4.1), suggesting that respondents perceive technological adoption as a significant contributor to improved organisational collaboration.

This consistency between qualitative and quantitative patterns underscores the robustness of the mixed-methods design.

Building on the descriptive overview, the next stage applied correlation and regression analysis to assess the statistical strength and direction of relationships between AI adoption, IoT implementation, cloud utilisation and collaboration effectiveness. The following tables present sequential analytical outputs from correlation to coefficient estimation.

Table 5 presents the correlation matrix between the core technology variables and collaboration effectiveness. All variables demonstrate moderate to strong positive relationships (r = 0.55–0.64). Cloud utilisation shows the strongest correlation with collaboration effectiveness (r = 0.64), followed by AI adoption (r = 0.62) and IoT implementation (r = 0.58). These results indicate that higher levels of technology adoption are associated with improved communication, coordination and knowledge sharing within energy sector teams.

**Table 5:** Correlation matrix.

Variables	1	2	3	4
1. Collaboration effectiveness	1.00			
2. AI Adoption (Q6)	0.62	1.00		
3. IoT implementation (Q7)	0.58	0.55	1.00	
4. Cloud utilisation (Q8)	0.64	0.59	0.60	1.00

The difference between *R*^2^ (0.437) and Adjusted *R*^2^ (0.421) reflects the adjustment for the number of predictors included in the regression model. The adjusted value provides a more conservative estimate of explained variance after accounting for model complexity.

The regression model explains approximately 43.7% of the variance in collaboration effectiveness (*R*^2^ = 0.437), with an adjusted *R*^2^ of 0.421 after controlling for the number of predictors included in the model. All three predictors were statistically significant at *p* < 0.01. Cloud utilisation demonstrated the strongest standardised effect (*β* = 0.421), followed by AI adoption (*β* = 0.412) and IoT implementation (*β* = 0.398). The overall model significance (*F* = 27.34, *p* < 0.001) confirms that the combined adoption of these technologies significantly predicts collaboration effectiveness within the UAE energy sector.

The regression outcomes were used to assess the six hypotheses presented in Section 2. The analysis confirmed that all three core technologies positively and significantly influence workplace collaboration. Organisational readiness and digital maturity emerged as partial moderating factors through qualitative insights, but were not separately tested in the quantitative phase. Similarly, generational resistance and uneven digital literacy were observed to have a dampening effect on adoption, partially supporting their hypothesised moderating role.

The quantitative analysis confirmed that innovative technologies significantly enhance collaborative efficiency across the UAE energy sector. The results demonstrated a strong and measurable association between technology adoption and collaboration effectiveness, with cloud computing showing the strongest predictive influence. These findings reinforce the qualitative insights and highlight that digital transformation driven by AI, IoT and cloud computing acts as a key enabler of collaborative innovation within energy organisations.

Each hypothesis was evaluated based on convergence between the interview themes and survey data. The results are summarised below:

*H1:* The adoption of AI positively influences workplace collaboration by enhancing real-time decision-making and coordination.

Supported. Qualitative data revealed that AI-driven analytics improved situational awareness and response time across operational teams, while survey results showed a strong positive correlation between AI utilisation and perceived decision-making efficiency. These effects are consistent with the UTAUT, particularly performance expectancy and facilitating conditions.

*H2*: The implementation of IoT systems positively influences workplace collaboration by enabling data integration across departments.

Supported. IoT-enabled data sharing was consistently associated with improved inter-departmental communication and faster maintenance coordination. These effects align with UTAUT’s construct of effort expectancy and facilitating conditions, reinforcing the role of technological integration in perceived ease of use and collaboration efficiency.

*H3:* The use of cloud computing platforms positively influences workplace collaboration by supporting remote accessibility and cross-functional knowledge sharing.

Supported. Both datasets confirmed that cloud-based platforms facilitated virtual collaboration, enhanced document version control and increased knowledge transparency. This finding reflects UTAUT’s emphasis on performance expectancy and facilitating conditions as drivers of collaborative adoption.

*H4:* Organisational readiness and digital maturity moderate the relationship between innovation technologies (AI, IoT, Cloud) and collaboration effectiveness.

Partially Supported. Organisations with advanced digital infrastructures demonstrated higher collaboration effectiveness; however, inconsistent implementation and uneven cultural adaptation suggest that readiness enhances but does not fully determine collaborative performance outcomes. This pattern corresponds with UTAUT’s recognition of facilitating conditions as necessary but not sufficient for sustained technology use.

*H5:* Generational resistance and uneven digital literacy negatively moderate the relationship between innovation technologies and collaboration outcomes.

Partially Supported. Thematic analysis revealed persistent skill and adoption disparities between senior and junior staff, while survey patterns indicated that digital literacy influences technology uptake but does not singularly explain variations in collaboration outcomes. This supports UTAUT’s social influence construct, illustrating how demographic and cultural dynamics shape adoption behaviour.

*H6:* The integration of AI, IoT and cloud computing fosters the emergence of new forms of cross-organisational collaboration networks that extend beyond traditional hierarchical structures in the UAE energy sector.

Supported. The findings demonstrated the formation of digitally networked teams across company boundaries, validating the emergence of non-hierarchical, project-driven collaboration models consistent with the Actor-Network Theory perspective.

#### 4.2.1 Summary of hypothesis testing

Four of the six hypotheses (H1, H2, H3, and H6) were fully supported, while H4 and H5 were partially supported. These outcomes underscore that technology adoption enhances collaboration when aligned with organisational readiness and workforce capability. Collectively, the findings are consistent with UTAUT, confirming that performance expectancy, effort expectancy, social influence and facilitating conditions collectively underpin effective digital collaboration in the UAE energy sector.

## 5 Discussion

This study examined how AI, IoT and cloud computing influence workplace collaboration in the UAE energy sector, addressing the central research question concerning how emerging digital technologies reshape organisational interaction and decision-making processes. The integration of qualitative and quantitative evidence demonstrates that these technologies significantly enhance collaboration by enabling real-time information sharing, predictive analytics and coordinated decision-making across organisational units. Instant data transfer and predictive analytics allow teams to respond proactively to operational challenges, reinforcing cross-functional collaboration across engineering, operations and digital management teams (Bojarajan et al., 2024). These findings are also consistent with Industry 4.0 literature, where digitalisation and electrification are recognised as core enablers of real-time data integration, advanced analytics and data-driven decision-making across industrial systems (Mubarak and Petraite, 2020; Sartono et al., 2024; Porter and Heppelmann, 2014). These results align with the findings of Neves et al. (2025) and Chwiłkowska-Kubala et al. (2023), who reported that digital transformation initiatives improve coordination efficiency by integrating technological systems with organisational processes. However, the present study extends these findings by demonstrating that in the UAE energy context digital technologies elevate collaboration from an operational necessity to a strategic organisational capability. These findings are also consistent with broader studies on digital transformation in industrial sectors, which highlight the role of integrated digital systems in enhancing coordination and organisational responsiveness within complex operational environments.

The findings also reinforce the theoretical expectations derived from the Unified Theory of Acceptance and Use of Technology. Performance expectancy, effort expectancy, social influence and facilitating conditions emerged as key drivers of technology acceptance, while TAM constructs of perceived usefulness and perceived ease of use functioned as underlying behavioural perceptions influencing adoption (cf. Mohd, 2024). Similar patterns were identified by Feng et al. (2025), who showed that cloud-based collaboration platforms gain higher acceptance when aligned with team-level objectives, and by Tantry et al. (2026), who emphasised that organisational support strengthens user adoption intentions within UTAUT frameworks. The present findings contribute to this literature by indicating that in complex industrial environments such as the UAE energy sector, technology adoption is driven not only by individual user perceptions but also by organisational collaboration demands, where digital tools become essential for coordinating multidisciplinary teams operating across geographically dispersed assets.

Notably, the two highest-ranked themes, technological impact and benefits of innovation technologies, align closely with TAM constructs of perceived usefulness and perceived ease of use. This reinforces that practical performance gains and perceived usability are the primary drivers of technology-supported collaboration in the UAE energy sector, while sustainability considerations, although important, exert a secondary influence.

Leadership commitment and organisational support were also found to be decisive factors in enabling successful digital adoption, addressing research questions related to organisational readiness and digital maturity. Prior studies have similarly emphasised the role of leadership in supporting technological change. Ali et al. (2024) demonstrated that leadership endorsement significantly increases technology uptake in energy organisations, while showed that cross-departmental governance mechanisms facilitate the implementation of digital initiatives. The findings of the present study support these arguments but extend them by highlighting that collaboration between departments, particularly between IT, engineering and sustainability divisions, acts as a multiplier of technology adoption outcomes. In the UAE context, where many energy organisations operate within hierarchical structures, coordinated leadership support appears essential for aligning digital transformation initiatives with operational and sustainability objectives.

From a strategic management perspective, the results contribute to the Resource-Based View by demonstrating that digitally enabled collaboration can function as a strategic organisational resource. Previous studies have shown that AI and IoT capabilities enhance organisational performance and competitive advantage (Bojarajan et al., 2024; Khalifa et al., 2025). Aguiar-Hernandez and Breetz (2024) further argued that digital infrastructure combined with skilled human capital creates organisational capabilities that are difficult for competitors to replicate. The present study builds upon these insights by demonstrating that the integration of digital technologies with collaborative work practices forms a distinctive organisational capability within UAE energy organisations. Rather than technology alone generating competitive advantage, the combination of technological infrastructure, skilled personnel and collaborative workflows creates a complex capability that strengthens operational agility and decision-making effectiveness.

The application of Actor-Network Theory provides an additional interpretive lens for understanding how these technological systems reshape organisational interaction. Consistent with Tantry et al. (2025), the findings indicate that digital technologies operate as active elements within socio-technical networks rather than as passive tools. Similarly argued that digital artefacts possess agency within organisational systems, while observed that automation technologies alter communication structures within Gulf-based industries. The present findings extend this perspective by demonstrating that in the UAE energy sector AI, IoT and cloud platforms actively structure collaboration by coordinating information flows, synchronising workflows and shaping decision-making rhythms across teams. In this sense, digital systems function as organisational actors that influence how collaboration emerges and evolves.

While the results strongly support the positive impact of digital technologies on collaboration, the analysis also revealed several contextual challenges. Consistent with prior research, cybersecurity concerns, legacy infrastructure and organisational resistance continue to limit the pace of digital transformation (Fasi, 2024; Mazhar et al., 2023). This also reflects broader concerns related to digital trust, particularly in environments where data is generated through interconnected IoT systems and shared across multiple interfaces. Trust in data integrity, system reliability and human–machine interaction is increasingly recognised as a critical factor influencing digital adoption in sensor-based and data-driven environments (Launer et al., 2022; Paliszkiewicz and Chen, 2022). However, this study identified an additional organisational barrier in the form of mid-level managerial hesitation toward technology-driven decision processes. Whereas previous studies often attribute resistance primarily to technical limitations or skill gaps, the findings suggest that structural inertia within management layers may slow the adoption of collaborative digital practices, particularly where established workflows are deeply embedded. Similar constraints have been widely reported in digital transformation literature, where organisational resistance, legacy system dependency and cybersecurity concerns are identified as persistent barriers to large-scale technology adoption in critical infrastructure sectors. Importantly, digital transformation in the energy sector should not be associated with workforce displacement due to skill gaps. Instead, prior research emphasises that targeted upskilling and reskilling initiatives are essential to enable employees to adapt to digital environments and support sustained organisational transformation (Paliszkiewicz and Chen, 2022). These findings also align with broader research on digital transformation, which highlights that organisational readiness requires a shift towards more agile, actor-oriented structures characterised by faster decision-making and reduced hierarchical constraints (Snow et al., 2017). In this context, organisations must make strategic choices regarding the depth of digital integration, whether through fully digitised operational solutions or through enhanced customer engagement models that position stakeholders as active participants in value creation.

The partial support observed for hypotheses relating to organisational readiness and generational resistance (H4 and H5) provides further insight into the UAE context. While organisations with higher levels of digital maturity demonstrated stronger collaborative outcomes, readiness alone did not fully determine adoption success. This suggests that even technologically advanced organisations must address cultural and structural factors when implementing digital transformation initiatives. Similarly, generational differences in digital literacy influenced perceptions of technology usefulness and ease of use but did not independently determine collaboration outcomes. These findings indicate that workforce diversity in skills and experience may influence adoption patterns but can be mitigated through training, organisational support and collaborative governance structures. The partial confirmation of these hypotheses therefore highlights the importance of institutional culture and leadership alignment within the UAE energy sector.

The findings also demonstrate how national policy frameworks accelerate digital transformation and collaborative innovation. Strategic initiatives such as the UAE Energy Strategy 2050 and the Net Zero by 2050 initiative create strong institutional incentives for adopting advanced digital technologies. Previous research has shown that UAE energy policy increasingly integrates digital innovation with sustainability objectives (Al-Hajri et al., 2024; Leal-Arcas, 2024). Crupi and Schiliro (2023) further observed that innovation-driven diversification policies stimulate investment in AI and IoT technologies across the Gulf region. The present study contributes additional insight by demonstrating that these technologies serve both operational and regulatory functions within UAE energy organisations. In addition to improving efficiency, digital platforms support sustainability monitoring, reporting and coordination between industry stakeholders and regulatory institutions. This is particularly relevant in the UAE context, where many energy organisations remain closely linked to hydrocarbon-based production systems. In such environments, digital technologies are not only tools for operational improvement but are essential for enabling the transition towards sustainability targets, including SDGs 7, 12 and 13. As highlighted in prior research, sustainability outcomes in energy-intensive industries are strongly dependent on improvements in operational efficiency, where digital innovation plays a critical enabling role (Karim et al., 2021; Nakandala et al., 2024).

An additional observation from the findings is the absence of explicit references to large-scale critical infrastructure failures or systemic energy disruptions. This is notable given the extensive literature identifying infrastructure vulnerability as a key risk within Gulf energy systems. One possible explanation is that many participants were primarily involved in operational, managerial or digital transformation roles rather than national infrastructure security functions. As a result, their perspectives may have focused more on organisational collaboration and technology adoption than on strategic infrastructure risk. Another possible explanation relates to the sensitivity of infrastructure security discussions within the energy sector, where employees may be reluctant to discuss vulnerabilities openly. Consequently, the absence of this theme should not be interpreted as evidence that such risks are insignificant, but rather as a reflection of the professional scope of the participants and the organisational contexts in which they operate.

The qualitative findings can be further interpreted through explicit alignment with established theoretical constructs. Theme 1 (technological impact on collaboration) reflects performance expectancy, as digital technologies enhance efficiency, coordination and decision-making outcomes. Theme 3 (benefits of innovation technologies) similarly reinforces perceived usefulness and effort expectancy, particularly through improvements in operational processes and ease of information access. Theme 2 (barriers to technological adoption) corresponds to facilitating conditions and social influence, where organisational readiness, trust and managerial support shape adoption behaviour. Theme 4 (technology and sustainability synergy) extends these constructs by illustrating how digital technologies operate within a broader digital ecosystem, integrating IoT, cloud computing and data-driven decision-making processes. This ecosystem perspective is consistent with prior research highlighting the interconnected role of digital technologies, data infrastructure and organisational capabilities in enabling Industry 4.0 transformation (Mubarak and Petraite, 2020).

Overall, the findings indicate that collaboration represents a central outcome of digital transformation in the UAE energy sector. AI, IoT and cloud computing technologies not only improve operational efficiency but also restructure organisational communication patterns, decision-making processes and strategic coordination. By integrating insights from UTAUT, TAM, RBV and Actor-Network Theory, the study demonstrates that digital technologies influence both behavioural adoption and organisational capability development. This integrated perspective helps explain how digital transformation initiatives generate broader organisational change within complex industrial environments.

The study offers three primary contributions to existing research. First, it provides empirical evidence from the UAE energy sector, a context that remains relatively underrepresented in global studies of digital transformation and technology adoption. Second, the findings extend the Resource-Based View by conceptualising collaboration itself as a strategic resource enabled by digital technologies. Third, by integrating Actor-Network Theory with technology adoption frameworks, the research demonstrates that digital systems function as active organisational actors that shape collaboration patterns, operational culture and sustainability governance.

Collectively, these findings enhance theoretical understanding of how digital transformation reshapes organisational collaboration within energy systems. They also offer practical implications for policymakers and industry leaders seeking to leverage digital technologies to support innovation, sustainability and organisational resilience within the UAE energy sector.

## 6 Conclusion

### 6.1 Realisation of the research objectives

The realisation of the research objectives is summarised in Table 6, which maps each objective to the key findings identified through the mixed-methods analysis.

**Table 6:** Realisation of research objectives.

Research objective	Key findings	Supporting evidence
Objective 1: Examine how AI, IoT and cloud computing influence workplace collaboration	Digital technologies enable real-time data sharing, predictive analytics and cross-functional coordination across organisational units	Themes 1 and 2; qualitative interviews and survey correlations
Objective 2: Identify barriers to technology adoption in the UAE energy sector	Cybersecurity concerns, legacy infrastructure, managerial resistance and workforce skill gaps limit full integration	Theme 2 findings and qualitative interview data
Objective 3: Evaluate the operational benefits of digital technologies	Technologies improve predictive maintenance, decision-making speed and operational efficiency	Themes 1 and 3; descriptive statistics and regression analysis
Objective 4: Assess the relationship between technology adoption and sustainability goals	Digital technologies support sustainability monitoring, renewable integration and policy compliance	Theme 4 findings and qualitative insights

The combined qualitative and quantitative findings demonstrate that AI, IoT and cloud computing significantly enhance collaborative practices across the UAE energy sector while also supporting operational efficiency and sustainability objectives (see Table 7).

**Table 7:** Regression analysis summary.

Model summary	Value	Model significance (ANOVA)	Value	Coefficients	*B*	Std. error	Beta	*t*	Sig.
*R*	0.661	Regression SS	24.912	Constant	1.234	0.543	—	2.27	0.026
*R* ^2^	0.437	Residual SS	32.051	AI Adoption (Q6)	0.321	0.089	0.412	3.61	0.001
Adjusted *R*^2^	0.421	Total SS	56.963	IoT Implementation (Q7)	0.278	0.082	0.398	3.39	0.001
Std. error of estimate	0.54	F	27.34	Cloud Utilisation (Q8)	0.305	0.091	0.421	3.36	0.001
		Sig.	0.000						

### 6.2 Contributions from the research

This research makes several theoretical, empirical and practical contributions.

First, it provides new empirical evidence from the UAE energy sector, a context that remains underrepresented in global digital transformation literature. By examining collaboration within a hydrocarbon-based economy undergoing digital and sustainability transitions, the study expands the geographic scope of technology adoption research.

Second, the study advances theoretical understanding by integrating UTAUT with TAM, RBV and Actor-Network Theory. This framework moves beyond individual adoption models by capturing how organisational resources and socio-technical networks shape collaborative outcomes.

Third, the findings contribute conceptually by demonstrating that digitally enabled collaboration can function as a strategic organisational resource. Within the RBV perspective, integrated digital ecosystems combining technology infrastructure and skilled human capital create capabilities that support competitive advantage.

Fourth, the study highlights the organisational barriers that remain within the UAE energy sector, particularly managerial inertia and structural resistance within mid-level management. This insight shifts the discussion from purely technical constraints to organisational dynamics influencing digital transformation.

Finally, the study contributes policy relevance by demonstrating how digital technologies support the implementation of national initiatives such as the UAE Energy Strategy 2050 and Net Zero by 2050, linking organisational collaboration directly to sustainability governance.

### 6.3 Policy-based contributions

The findings provide several implications for policymakers supporting digital transformation in the UAE energy sector.

First, policymakers should establish a national digital energy skills accreditation framework by 2026 in partnership with the Ministry of Energy and Infrastructure, the UAE AI Council and higher education institutions. Such a framework could standardise training in AI literacy, cloud security and IoT systems across the energy workforce.

Second, the federal government could introduce performance-linked digital transformation incentives for energy firms adopting interoperable data platforms aligned with national sustainability reporting frameworks.

Third, national regulators should develop secure inter-agency digital infrastructure standards that enable energy utilities and government institutions to share operational data safely while maintaining cybersecurity safeguards.

### 6.4 Practical contributions

Energy organisations should prioritise the development of integrated cloud-based collaboration platforms that allow engineering, IT and sustainability teams to operate from shared real-time data environments.

Companies should also implement modular digital transformation strategies, allowing legacy infrastructure to be upgraded incrementally rather than through disruptive full-system replacements.

Additionally, organisations should establish cross-functional digital transformation teams composed of engineers, data analysts and sustainability specialists to coordinate technology adoption and accelerate collaborative decision-making.

### 6.5 Limitations

Several limitations should be acknowledged.

First, the qualitative interviews primarily involved professionals from government-owned or semi-private energy organisations in Abu Dhabi and Dubai. This may limit the generalisability of the findings to smaller private-sector companies or energy organisations operating in other regions.

Second, most participants held mid-level or senior positions. While this provided strategic perspectives on digital transformation, the study may underrepresent the experiences of frontline technical staff who interact directly with operational systems.

Third, the study relied partly on self-reported perceptions of technology adoption and collaboration outcomes, which introduces the possibility of self-report bias and common method variance. Participants may have reported favourable outcomes due to organisational expectations or perceived technological benefits.

Fourth, the research adopted a cross-sectional design, capturing perceptions at a single point in time. As digital transformation initiatives evolve rapidly, longitudinal studies would provide deeper insight into how collaborative practices develop as technology maturity increases.

Another limitation relates to the broader geopolitical context within which the UAE energy sector operates. While this study examined organisational and technological barriers to digital collaboration, external disruptions such as geopolitical tensions, international conflicts and regional instability may also influence the adoption and implementation of digital technologies. Energy infrastructure and supply chains in the Middle East are particularly sensitive to such disruptions, which can affect investment priorities, cybersecurity strategies and cross-border energy cooperation. As a result, the findings of this research should be interpreted within the relatively stable institutional context of the UAE, and future disruptions of a geopolitical nature may alter the trajectory of digital transformation within the sector.

Another limitation relates to the composition of the participant sample. Most respondents were drawn from large, technologically advanced organisations such as ADNOC, DEWA and TAQA. These institutions possess significant financial resources, mature digital infrastructure and strong policy alignment with national innovation initiatives. Consequently, the findings may reflect the experiences of leading organisations within the UAE energy sector rather than the full spectrum of firms operating in the industry. Smaller companies or emerging renewable startups with more limited technological capacity may face different adoption challenges and collaboration dynamics.

Finally, the UAE’s unique institutional context, including strong government support for digital innovation and sustainability policy, may limit the direct transferability of these findings to countries with different regulatory environments or technological capacities.

### 6.6 Recommendations

Based on the findings, several targeted recommendations are proposed.

First, UAE policymakers should implement a national digital workforce certification programme for energy professionals by 2026, focusing on AI operations, IoT integration and cloud cybersecurity.

Second, energy organisations should develop integrated digital collaboration platforms that connect operational data from sensors, predictive analytics systems and sustainability reporting dashboards.

Third, organisations should introduce structured change management programmes to address managerial resistance and improve digital adoption across senior and mid-level leadership.

Fourth, partnerships between energy companies, universities and technology providers should be expanded to create professional certification pathways in digital energy technologies, ensuring workforce capabilities evolve alongside technological infrastructure.

### 6.7 Future research agenda

Future research should explore how collaboration evolves as digital technologies mature across energy organisations. Longitudinal studies could examine the long-term organisational impacts of AI, IoT and cloud integration, particularly regarding workforce adaptation and governance structures.

Future studies may also examine how geopolitical instability, regional conflicts and global energy market disruptions influence digital transformation strategies and collaborative practices within Middle Eastern energy organisations.

Comparative studies across Gulf Cooperation Council countries may also provide insight into how regulatory environments and policy frameworks influence digital adoption in energy sectors. Finally, future research could investigate how procurement strategies, vendor partnerships and digital infrastructure investment decisions shape the success of large-scale digital transformation initiatives.

## Data Availability

The original contributions presented in the study are included in the article/supplementary material, further inquiries can be directed to the corresponding author.
