# Supplementary material for: Innovative technologies and workplace collaborations in the energy sector based in the United Arab Emirates
Source: Front Artif Intell. 2026 Apr 22;9:1798647. doi: 10.3389/frai.2026.1798647 (PMC13144080; doi:10.3389/frai.2026.1798647)
Supplement: Supplementary file 1 [file Supplementary_file_1.docx]

# Appendix A (Transcript Excerpt with Manual Coding)

**Participant ID:**  P7
**Role:**  Digital Transformation Lead, Utility Provider
**Date of Interview:**  15 January 2025
**Method:**  Semi-structured interview via Microsoft Teams
**Duration:**  22 minutes

**Excerpt:**

| **Transcript Segment** | **Initial Code** | **Theme** |
| --- | --- | --- |
| “We're used to the old system. Switching feels like a risk, not an upgrade.” | Change Aversion | Resistance to Change |
| “We’ve tried new platforms, but every department ends up using its own. It’s chaotic.” | System Fragmentation | Cloud Integration Challenges |
| “Field staff don’t have proper training to use IoT-enabled dashboards. They just ignore them.” | Skills Gap | Workforce Digital Readiness |
| “The AI-generated reports are great, but upper management doesn’t trust the outputs yet.” | Trust in AI | AI-Augmented Decision Making |
| “We waste a lot of time copying data from one tool to another because there’s no compatibility.” | Interoperability Issues | Legacy System Limitations |

**Notes:**

- The participant demonstrated a strong awareness of integration and digital capability issues.
- Their responses reflected a dual theme tension between technological advancement and organisational inertia.
- Manual coding was applied using colour-coded highlights in the transcript, later transcribed into this summary table.

# Appendix B (Interview Data Summary Table)

| Participant ID | Role | Years of Energy Experience | Main Theme | Supporting Quote |
| --- | --- | --- | --- | --- |
| P1 | Operations Manager, National Energy Firm | 16 | Resistance to Change | Connectivity in remote areas is still poor, making real-time data sharing unreliable. |
| P2 | Senior Engineer, Renewable Energy Division | 23 | Interdepartmental Data Sharing | Connectivity in remote areas is still poor, making real-time data sharing unreliable. |
| P3 | ICT Specialist, Oil & Gas Corporation | 12 | Cybersecurity and Compliance | There's no standard format. Data from one unit can't be used by another. |
| P4 | Maintenance Supervisor, Offshore Operations | 19 | Workforce Digital Readiness | Decisions are now backed by data, not just gut feeling. |
| P5 | Digital Transformation Lead, Utility Provider | 19 | Interdepartmental Data Sharing | Without strong leadership support, tech changes stall. |
| P6 | Project Manager, Energy Transition Unit | 13 | Resistance to Change | We need real-time access, but our legacy tools don’t support that. |
| P7 | SCADA Systems Analyst, Grid Control Centre | 24 | Collaboration Enhancement via AI | AI has helped us cut down our decision cycles by days. |
| P8 | Cybersecurity Specialist, Oil Refinery | 5 | Workforce Digital Readiness | Old systems are incompatible with most of the new solutions. |
| P9 | Innovation Officer, Solar Energy Plant | 9 | IoT-Driven Maintenance Optimisation | AI has helped us cut down our decision cycles by days. |
| P10 | Field Engineer, Remote Oil Field | 5 | Legacy System Limitations | There's no standard format. Data from one unit can't be used by another. |
| P11 | Regulatory Compliance Officer, Energy Authority | 22 | Interdepartmental Data Sharing | We need tools that work offline too, given our field constraints. |
| P12 | Strategy Consultant, Clean Energy Projects | 22 | Legacy System Limitations | Multiple platforms are complex to integrate. Each team prefers their own system. |
| P13 | Asset Integrity Coordinator, Offshore Rigs | 16 | Cloud Integration Challenges | We need real-time access, but our legacy tools don’t support that. |
| P14 | AI Solutions Architect, Smart Grid Team | 21 | Cybersecurity and Compliance | Multiple platforms are complex to integrate. Each team prefers their own system. |
| P15 | Data Analyst, Energy Demand Planning | 17 | IoT-Driven Maintenance Optimisation | We're rolling out AI, but the field teams aren’t involved early enough. |
| P16 | HSE Manager, Oil & Gas Operations | 17 | Digital Infrastructure Gaps | We're rolling out AI, but the field teams aren’t involved early enough. |
| P17 | Power Systems Engineer, Utility Firm | 15 | Legacy System Limitations | Cross-departmental collaboration has improved since we adopted shared dashboards. |
| P18 | Blockchain Coordinator, Smart Contracts Group | 14 | Interdepartmental Data Sharing | Security protocols have not caught up with our cloud migration. |
| P19 | Training Manager, Technical Development Centre | 8 | Workforce Digital Readiness | Compliance is slowing down tech adoption. We need better alignment. |
| P20 | Cloud Platform Engineer, Energy IT Department | 13 | Collaboration Enhancement via AI | Security protocols have not caught up with our cloud migration. |

# Appendix C (Thematic Map)

| **Theme** | **Sub-theme** |
| --- | --- |
| Resistance to Change | Generational Divide |
| Resistance to Change | Job Security Concerns |
| Digital Infrastructure Gaps | Connectivity Issues |
| Digital Infrastructure Gaps | Legacy System Constraints |
| AI-Augmented Decision Making | Faster Decision Cycles |
| AI-Augmented Decision Making | Scepticism Toward AI |
| IoT-Driven Maintenance Optimisation | Predictive Maintenance |
| IoT-Driven Maintenance Optimisation | Sensor Integration |
| Cloud Integration Challenges | Platform Compatibility |
| Cloud Integration Challenges | Remote Access Limitations |
| Workforce Digital Readiness | Lack of Training |
| Workforce Digital Readiness | Technology Aversion |
| Cybersecurity and Compliance | Data Protection Issues |
| Cybersecurity and Compliance | Policy Misalignment |
| Legacy System Limitations | Incompatibility with Modern Tech |
| Legacy System Limitations | Maintenance Burden |
| Interdepartmental Data Sharing | Lack of Standardisation |
| Interdepartmental Data Sharing | Communication Gaps |
| Collaboration Enhancement via AI | Shared Dashboards |
| Collaboration Enhancement via AI | Improved Coordination |

# Appendix D (Codebook Excerpt)

| **Theme** | **Sub-theme** | **Definition** | **Example Quote** |
| --- | --- | --- | --- |
| Resistance to Change | Generational Divide | Older staff are less receptive to digital tools | Not everyone is ready to embrace digital tools; we need more training. |
| Cloud Integration Challenges | Platform Compatibility | Difficulty in integrating diverse digital systems | Multiple platforms are complex to integrate. Each team prefers their own system. |
| AI-Augmented Decision Making | Faster Decision Cycles | Use of AI to speed up decision-making | AI has helped us cut down our decision cycles by days. |
| IoT-Driven Maintenance Optimisation | Predictive Maintenance | Using IoT to anticipate maintenance needs | With IoT, we can now predict equipment failures before they happen. |
| Digital Infrastructure Gaps | Connectivity Issues | Inadequate network support for field operations | Connectivity in remote areas is still poor, making real-time data sharing unreliable. |

# Appendix E (Coding Sheet Excerpt)

| **Transcript Line** | **Assigned Code** |
| --- | --- |
| We’re used to the old system. Switching feels like a risk, not an upgrade. | Resistance to Change |
| Connectivity in remote areas is still poor. | Digital Infrastructure Gaps |
| AI has helped us cut down our decision cycles by days. | AI-Augmented Decision Making |
| Multiple platforms are complex to integrate. | Cloud Integration Challenges |
| With IoT, we can now predict equipment failures before they happen. | IoT-Driven Maintenance Optimisation |

# Appendix F (Consent Form)

**Informed Consent Form**

**Doctor of Business Administration (DBA) Research Study**

**Title:**  *Innovation Technologies and Workplace Collaboration in the UAE Energy Sector*
**Researcher:**  Jack Charles Boath (Doctorate Candidate).
**Institution:**  Swiss School of Management and Kings Business School FZC LLC

**Purpose of the Study:**

This research explores how innovative technologies such as AI, IoT and cloud computing influence workplace collaboration within the UAE energy sector.

**Participation Details:**

Your participation involves a 30-minute recorded interview.

You may refuse to answer any question or withdraw at any time without penalty.

Your responses will be anonymised and kept strictly confidential.

**Confidentiality & Data Use:**

All information will be stored securely and used solely for academic purposes.

Identifiers such as your name, job title or organisation will not appear in any published materials.

Data will be stored securely on Microsoft Azure cloud servers (UAE region) for a period of 5 years.

**Voluntary Participation:**

Your participation is entirely voluntary. There are no risks or direct benefits from participating and you are free to decline or withdraw at any stage.

**Consent Declaration (Please Tick):**

☐ I have read and understood the information above.
☐ I voluntarily agree to participate in this study.
☐ I understand that my identity will be protected and my responses kept confidential.
☐ I consent to the audio recording of my interview for transcription and analysis.

| **Participant Name:** | [Full Name] | **Researcher Name:** | Jack Charles Boath |
| --- | --- | --- | --- |
| **Participant Signature:** | [Sign] | **Researcher Signature:** | [Sign] |
| **Date:** | [DD/MM/YYYY] | **Date:** | [DD/MM/YYYY] |

# Appendix G (Interview Guide)

The semi-structured interview aims to explore the impact of digital technologies (AI, IoT, cloud solutions) on collaboration, operational efficiency and workforce readiness in the UAE energy sector.

Interviewer to begin with an introduction and explain the purpose of the study, reassure participants about confidentiality and voluntary participation, use open-ended questions to encourage detailed responses and follow up with probes as necessary for clarification.

Section 1: General Background

1. Can you describe your current role and responsibilities in the organisation?
2. How long have you been working in the energy sector?

Section 2: Technology Adoption and Change Management

1. How does your organisation typically respond to the introduction of new digital technologies?
2. What factors influence acceptance or resistance to change among employees?
3. Do generational differences impact the adoption of new technologies?

Section 3: Digital Infrastructure and System Integration

1. What are the biggest challenges related to digital infrastructure (e.g., connectivity in remote areas)?
2. How do legacy systems affect your workflow and digital transformation efforts?
3. Have you faced any compatibility issues when integrating new platforms or systems?

Section 4: AI and Decision-Making

1. Are AI-driven tools used in your department? If yes, in what capacity?
2. How confident are you in the outputs generated by AI tools?
3. Has AI impacted decision-making speed and accuracy in your work?

Section 5: IoT and Predictive Maintenance

1. How is IoT currently being utilised for operations and maintenance in your organisation?
2. Do predictive maintenance tools reduce downtime or improve efficiency?
3. What barriers exist to wider IoT adoption?

Section 6: Workforce Readiness and Training

1. How prepared is your workforce to use new digital tools?
2. What types of training or upskilling programs are available?
3. Do you think there is technology aversion among specific groups or teams?

Section 7: Cybersecurity and Compliance

1. How do compliance and cybersecurity requirements affect digital adoption?
2. What security challenges have you experienced with cloud migrations or digital systems?

Section 8: Collaboration and Data Sharing

1. How effective is data sharing across departments?
2. Has AI improved collaboration between teams?
3. What digital tools have had the most significant impact on improving coordination?

Closing Question:

1. Is there anything else you would like to share regarding the challenges or benefits of digital transformation in your organisation?

# Appendix H (Survey)

# Title:

Impact of Digital Technologies on Collaboration and Operational Efficiency in the UAE Energy Sector

# Purpose Statement:

This survey is designed to understand how emerging technologies (AI, IoT, Cloud Computing) influence collaboration, operational efficiency and workforce readiness in the UAE energy sector. Your responses will remain confidential and will be used solely for academic purposes.

## Section A: Demographics

1. Gender:

☐ Male
☐ Female
☐ Prefer not to say

2. Age Group:

☐ 20–29
☐ 30–39
☐ 40–49
☐ 50+

3. Years of Experience in the Energy Sector:

☐ Less than 5
☐ 5–10
☐ 11–15
☐ 16+

4. Current Role:

☐ Operations
☐ IT/Technology
☐ Leadership/Management
☐ Other

5. Type of Organisation:

☐ Oil & Gas
☐ Renewable Energy
☐ Utilities
☐ Other

##

## Section B: Technology Adoption and Collaboration

Use a 5-point Likert scale:
(1 = Strongly Disagree | 2 = Disagree | 3 = Neutral | 4 = Agree | 5 = Strongly Agree)

6. My organisation encourages the adoption of new digital technologies.

7. Employees are open to learning and using new technologies.

8. Leadership actively supports digital transformation initiatives.

9. Generational differences create challenges in technology adoption.

## Section C: AI and IoT Usage

10. AI tools have improved decision-making in my organisation.

11. I trust the outputs generated by AI-based tools.

12. IoT solutions (e.g., predictive maintenance) have reduced downtime.

13. Integration of AI and IoT systems enhances operational efficiency.

## Section D: Cloud and Data Integration

14. Cloud platforms have improved collaboration across departments.

15. Integration between legacy and modern systems is seamless.

16. Data sharing between teams is efficient and reliable.

##

## Section E: Workforce Readiness and Training

17. My organisation provides adequate training for digital technologies.

18. Employees feel confident using new digital tools.

19. Technology aversion is common among certain employee groups.

## Section F: Cybersecurity and Compliance

20. Cybersecurity requirements delay the adoption of new technologies.

21. Compliance regulations increase the complexity of digital transformation.

22. Security concerns limit the adoption of cloud-based platforms.

## Section G: Overall Impact

23. Digital technologies have improved collaboration within my organisation.

24. Digital technologies have enhanced overall operational performance.

25. The benefits of adopting digital technologies outweigh the challenges.

**Thank you for your participation!**
